# Longitudinal development of incident gout from low-normal baseline serum urate concentrations: individual participant data analysis

**DOI:** 10.1186/s41927-021-00204-4

**Published:** 2021-08-28

**Authors:** Philip C. Robinson, Christopher Frampton, Amanda Phipps-Green, Tuhina Neogi, Lisa Stamp, William Taylor, Tony R. Merriman, Nicola Dalbeth

**Affiliations:** 1 University of Queensland Faculty of Medicine, Herston, Herston, Queensland Australia; 2 Royal Brisbane and Women’s Hospital, Metro North Hospital and Health Service, Herston, Queensland Australia; 3 Department of Medicine, University of Otago, Christchurch, New Zealand; 4 Department of Biochemistry, University of Otago, Dunedin, New Zealand; 5 Department of Medicine, Boston University School of Medicine, Boston, USA; 6 Department of Medicine, University of Otago, Wellington, New Zealand; 7 Division of Clinical Immunology and Rheumatology, University of Alabama at Birmingham, Birmingham, USA; 8 Department of Medicine, University of Auckland, Auckland, New Zealand

**Keywords:** Gout, Incidence, Epidemiology, Incident, Urate, Diagnosis

## Abstract

**Introduction:**

Elevated serum urate (SU) concentration is the central risk factor for the development of gout. The aim of this study was to examine the incidence of gout in people with low and normal SU levels (< 7.00 mg/dL).

**Methods:**

Longitudinal cohort data from the Atherosclerosis Risk in Communities Study (ARIC), Coronary Artery Risk Development in Young Adults Study (CARDIA), and both the Original and Offspring cohorts of the Framingham Heart Study (FHS) were used to determine incident gout by baseline SU over 3, 5, 10, 12 and 15 year periods. A Cox proportional hazards model with covariables of age, gender, ethnicity, and cohort was calculated to report the hazard ratios (HR) for incident gout.

**Results:**

The incidence of gout at 15 years for a baseline SU < 4.00 mg/dL was 0.59%, 4.00–4.49 mg/dL was 1.28%, 4.50–4.99 mg/dL was 0.86%, 5.00–5.49 mg/dL was 0.94%, 5.50–5.99 mg/dL was 1.52%, 6.00–6.49 mg/dL was 2.91%, 6.50–6.99 mg/dL was 3.2%, and > 7.00 mg/dL was 12.2%. In an adjusted Cox proportional hazards model, compared to the referent baseline SU < 4.00 mg/dL, there was a non-significant increase in incident gout for baseline SU bands between 4.00–5.49 mg/dL, whereas incident gout was significantly increased for SU 5.50–5.99 mg/dL (HR 2.60), 6.00–6.49 mg/dL (HR 3.70), 6.50–6.99 mg/dL (HR 5.24) and > 7.00 mg/dL (HR 18.62).

**Conclusion:**

A baseline SU of 5.50 mg/dL or more is a risk factor for development of gout over 15 years. However, incident gout does occur over time in a small proportion of people with lower baseline SU levels.

**Supplementary Information:**

The online version contains supplementary material available at 10.1186/s41927-021-00204-4.

Many longitudinal observational studies have demonstrated that the risk of developing gout is strongly related to serum urate level, with the incidence of gout increasing with higher serum urate levels [1–5]. For example, the Normative Aging Study reported that the incidence of gout with a serum urate < 6.0 mg/dL was 0.8/1000 years, increasing to 4.1/1000 years with a serum urate of 7.0–7.9, and rising dramatically to 70.2/1000 years in those with a serum urate ≥10 mg/dL [1]. Modifiable and non-modifiable factors influence serum urate including body weight, medications, genetics, systemic inflammation, and to a small degree diet [6–12]. Dietary and genetic factors may interact and modify risk further [13, 14]. Current guidelines almost universally recommend a treatment target of < 6.00 mg/dL for the treatment of gout [15]. While direct evidence of the validity of this particular target is lacking it is known that reducing serum urate below this level reduces gout flares over time [16].

While previous studies have shown the relationship between elevated serum urate and incident gout, the incidence of gout over a prolonged follow-up period is not well described for people with low to normal serum urate [1–4]. The concept of ‘low’ and ‘normal’ serum urate depends on the clinical context. Values for these concepts are not defined and is relative to both gender and co-morbidities, for example kidney function. Exploring the incidence of gout for a wide range of serum urate levels is important to understand the risk of gout for the majority of the population, who do not have elevated serum urate levels.

The aim of this study was to define the rate of incident clinically evident gout in those with low and normal serum urate levels (< 7.00 mg/dL).

## Methods

### Inclusion

The cohorts and participant characteristics from the Atherosclerosis Risk in Communities Study (ARIC), Coronary Artery Risk Development in Young Adults Study (CARDIA), and both the Original and Offspring cohorts of the Framingham Heart Study (FHS) have been described previously [3]. Briefly, longitudinal observational cohort studies with (a) publicly available individual-level data, (b) incident gout data available and (c) clinically evident gout defined using recognised classification criteria, or doctor diagnosis, or participant self-report of disease, or self-report of doctor diagnosis, and (d) serum urate measured prior to assessment for incident gout; and (e) a minimum follow-up period of 3 years were included [17]. Database of Genotype and Phenotype project #834 (The genetic basis of gout) was utilised to access individual participant-level data.

### Outcomes

The outcome of the study was incident gout. For all the included studies baseline data was taken as the first study visit where there was information about serum urate and the presence or absence of gout. Participants with baseline gout were excluded so the analysis time period in each study started with all participants without gout. The diagnosis of gout was then progressively recorded when it occurred in the participants of each study. The actual date was used preferably but if not the timepoint when it was next recorded. Then the incidence of gout was calculated over time for the timepoints 3 years, 5 years, 10 years, 12 years and 15 years. The number of incident cases was calculated at each timepoint and then the cumulative incidence as a percentage was also calculated.

### Analyses

The upper limit of baseline serum urate for analysis was selected as 7.00 mg/dL, as this value is frequently used to defined hyperuricaemia in research settings [18]. This value is also close to the crystallization point of urate at temperature 37 °C in laboratory studies (~ 6.8 mg/dL) [19]. Cox proportional hazards (CPH) models were constructed using < 4.00 mg/dL as the referent baseline serum urate level. The serum urate bands of 0.50 mg/dL were constructed up to and including 7.00 mg/dL, and then an additional category greater than 7.00 mg/dL was also added as a comparator. The CPH models assessed the incidence of gout at any time during follow-up. The CPH models were built with the covariables age, ethnicity and cohort, in addition to sex for the entire cohort analysis. The CPH models were completed in all participants, males, females, and in exploratory analysis, the female group was divided into a ≤ 50 years of age at the start of the observation period and > 50 years of age at the start of the observation period to take into account the increase in serum urate that occurs around the time of menopause [20]. To provide a widely useable tool for modelling in subsequent projects models were constructed to fit the data. Dependent on the distribution either a linear or an exponential model was created to provide a best fit for the underlying data at each time point: 3, 5, 10, 12 and 15 years. No imputation of missing data was used. All analyses were undertaken using the Statistical Package for Social Sciences V.24.0 software.

### Ethical approval

All participants gave written informed consent. The component studies (ARIC, FHS, CARDIA) had separate IRB approvals, described at www.ncbi.nlm.nih.gov/gap. This study was approved by the University of Otago Human Health Ethics Committee.

## Results

### Baseline serum urate levels

The assembled cohort included 18,889 participants who were gout-free at baseline, with mean (SD) 11.2 (4.2) years and 212,363 total participant-years of follow-up (Table 1 and Supplementary Table 1). There were 11,667/18,889 (61.8%) participants with a baseline serum urate < 6.00 mg/dL, and 15,669/18,889 (83.0%) participants with a baseline serum urate < 7.00 mg/dL. Baseline serum urate levels were lower in females than males; 8386/10,609 (78%) females and 3261/8280 (39%) males had a baseline serum urate of < 6.00 mg/L, and 9763/10,609 (92%) females and 5906/8280 (71%) male had a baseline serum urate < 7.00 mg/dL.

**Table 1:** Baseline data of included participants

	N	Mean (SD) Age at Baseline	Ethnicity: White or Caucasian	Ethnicity: Other	Mean (SD) Urate (mg/dL)
ARIC (Male)	4558	54.3 (5.7)	3770 (82.7)	788 (17.3)	6.6 (1.3)
ARIC (Female)	5814	53.6 (5.6)	4433 (76.2)	1381 (23.8)	5.4 (1.3)
ARIC (Total)	10,372	53.9 (5.7)	8203 (79.1)	2169 (20.9)	5.9 (1.5)
CARDIA (Male)	1520	25 (3.6)	870 (57.2)	650 (42.8)	6.2 (1.2)
CARDIA (Female)	1942	25.1 (3.7)	1001 (51.5)	941 (48.5)	4.5 (1.0)
CARDIA (Total)	3462	25.1 (3.6)	1871 (54)	1591 (46)	5.2 (1.3)
FHS Original (Male)	1027	41.6 (9.3)	1012 (98.5)	15 (1.5)	5.9 (1.2)
FHS Original (Female)	1223	42.1 (9.1)	1204 (98.4)	19 (1.6)	4.2 (1.0)
FHS Original (Total)	2250	41.9 (9.2)	2216 (98.5)	34 (1.5)	4.9 (1.3)
FHS Offspring (Male)	1175	65.3 (7.6)	1175 (100)	0 (0)	5.9 (1.2)
FHS Offspring (Female)	1630	66.3 (7.9)	1630 (100)	0 (0)	4.9 (1.3)
FHS Offspring (Total)	2805	65.9 (7.8)	2805 (100)	0 (0)	5.2 (1.3)
Overall (Male)	8280	48.9 (14.3)	6827 (82.5)	1453 (17.5)	6.4 (1.3)
Overall (Female)	10,609	49 (14.4)	8268 (77.9)	2341 (22.1)	5.0 (1.3)
Overall (Total)	18,889	49 (14.3)	15,095 (79.9)	3794 (20.1)	5.5 (1.5)

Abbreviation: *SD* Standard deviation

### Gout incidence

As previously reported [3], the cumulative incidence of gout by 15 years was 3.2% (95% CI, 2.8 to 3.6%) in the entire cohort, with higher cumulative incidence in males than females; 4.94% (95% CI, 4.34 to 5.54%) in males and 1.90% (95% CI, 1.58 to 2.2%) in females. In the entire cohort, for the time periods 3 years, 5 years, 10 years and 12 years there was no clear pattern of increase in incident gout until 6 mg/dL, and then incidence clearly increased from the lower urate levels and dramatically increased in the > 7 mg/dL band (Table 2). The incidence of gout at 15 years with a baseline serum urate < 4.00 mg/dL was 0.59% (Table 2). For subsequent urate bands between 4.00 mg/dL to 5.99 mg/dL, there was a largely static incidence of gout. For participants with baseline serum urate of 6.00 mg/dL to 7.00 mg/dL the incidence rose more sharply to 2.9% for 6.00–6.49 mg/dL and 3.2% for participants with baseline serum urate of 6.50–6.99 mg/dL. For participants with baseline serum urate > 7.00 mg/dL, the 15 year incidence was 12.2%. This pattern was also observed in the separate analyses of males and females (Table 2), as well as in females > 50 years old at the start of the observation period (Supplementary Table 2).

**Table 2:** Raw incidence of gout at timepoints 3, 5, 10, 12 and 15 years of follow-up in the cohort in combined cohort, males and females

	Serum urate (mg/dL)	N	3 Years	5 Years	10 Years	12 Years	15 Years
**All**	< 4.00	2490	0.08%	0.20%	0.46%	0.52%	0.59%
	4.00–4.49	1896	0.27%	0.32%	0.71%	0.94%	1.28%
	4.50–4.99	2383	0.13%	0.30%	0.65%	0.75%	0.86%
	5.00–5.49	2535	0.16%	0.24%	0.83%	0.94%	0.94%
	5.50–5.99	2363	0.13%	0.34%	0.93%	1.27%	1.52%
	6.00–6.49	2192	0.28%	0.51%	1.33%	1.73%	2.91%
	6.50–6.99	1810	0.22%	0.45%	1.96%	2.81%	3.20%
	≥7.00	3220	1.44%	3.41%	7.95%	10.44%	12.22%
**Males**	< 4.00	202	0.53%	1.61%	2.25%	2.25%	2.25%
	4.00–4.49	291	0.00%	0.35%	1.09%	1.74%	2.41%
	4.50–4.99	594	0.51%	0.51%	1.06%	1.06%	1.41%
	5.00–5.49	968	1.41%	1.41%	0.57%	0.80%	0.80%
	5.50–5.99	1226	0.17%	0.42%	0.93%	1.31%	1.71%
	6.00–6.49	1365	0.37%	0.74%	1.44%	1.80%	3.35%
	6.50–6.99	1260	0.32%	0.56%	1.97%	3.03%	3.50%
	≥7.00	2374	1.66%	3.80%	7.87%	9.84%	11.38%
**Females**	< 4.00	2288	0.04%	0.09%	0.32%	0.38%	0.45%
	4.00–4.49	1605	0.25%	0.32%	0.64%	0.78%	1.06%
	4.50–4.99	1789	2.31%	0.23%	0.52%	0.66%	0.66%
	5.00–5.49	1567	0.26%	0.39%	0.99%	0.99%	0.99%
	5.50–5.99	1137	0.09%	0.27%	0.92%	1.21%	1.21%
	6.00–6.49	827	0.12%	0.12%	1.15%	1.72%	1.72%
	6.50–6.99	550	2.44%	0.19%	1.97%	1.97%	1.97%
	≥7.00	846	0.84%	2.30%	8.19%	13.06%	16.45%

### Cox proportional hazards models

In the entire cohort, compared to the referent baseline serum urate < 4.00 mg/dL, there was no significant increased risk of incident gout for baseline serum urate bands up to 5.49 mg/dL. However, the 4.00–4.49 group had a hazard ratio of 1.96 with a 95% CI of 1.00–3.83, suggesting a trend to increased gout in this urate band. Incident gout risk was significantly increased in those with baseline serum urate 5.50–5.99 mg/dL (HR 2.60), 6.00–6.50 mg/dL (HR 3.70), 6.50–6.99 mg/dL (HR 5.24) and > 7.00 mg/dL (HR 18.62) (Table 3). The full model adjusted with covariables and unadjusted is shown in Supplementary Table 3.

**Table 3:** Cox proportional hazards models for the entire group, males, and females (adjusted for the following covariates: age, ethnicity and original cohort, in addition to gender for the entire group analysis)

	Adjusted models						
	Serum urate	Beta	SE	P	Hazard ratio	95% Lower CI	95% Upper CI
	Referant < 4.0 mg/dL						
**All**	4.0–4.49 mg/dL	0.671	0.343	0.050	1.96	1.00	3.83
	4.5–4.99 mg/dL	0.357	0.347	0.304	1.43	0.72	2.82
	5.0–5.49 mg/dL	0.502	0.335	0.134	1.65	0.86	3.19
	5.5–5.99 mg/dL	0.957	0.317	0.003	2.60	1.40	4.85
	6.0–6.49 mg/dL	1.308	0.308	0.000	3.70	2.02	6.77
	6.5–6.99 mg/dL	1.657	0.305	0.000	5.24	2.88	9.53
	≥7.00 mg/dL	2.924	0.279	0.000	18.62	10.78	32.15
**Male**	4.0–4.49 mg/dL	0.068	0.646	0.916	1.07	0.30	3.80
	4.5–4.99 mg/dL	−0.381	0.627	0.543	0.68	0.20	2.33
	5.0–5.49 mg/dL	−0.767	0.628	0.222	0.46	0.14	1.59
	5.5–5.99 mg/dL	0.119	0.551	0.829	1.13	0.38	3.32
	6.0–6.49 mg/dL	0.593	0.532	0.264	1.81	0.64	5.13
	6.5–6.99 mg/dL	0.922	0.528	0.081	2.51	0.89	7.07
	≥7.00 mg/dL	2.105	0.508	0.000	8.21	3.04	22.20
**Female**	4.0–4.49 mg/dL	0.649	0.406	0.110	1.91	0.86	4.25
	4.5–4.99 mg/dL	0.369	0.422	0.381	1.45	0.63	3.31
	5.0–5.49 mg/dL	0.819	0.399	0.040	2.27	1.04	4.95
	5.5–5.99 mg/dL	0.981	0.412	0.017	2.67	1.19	5.98
	6.0–6.49 mg/dL	1.052	0.438	0.016	2.86	1.21	6.76
	6.5–6.99 mg/dL	1.422	0.439	0.001	4.14	1.75	9.80
	≥7.00 mg/dL	2.958	0.345	0.000	19.27	9.80	37.87

Abbreviations: *SE* Standard error, *CI* Confidence Interval

In males, compared to the referent baseline serum urate < 4.00 mg/dL, there was no clear increased risk in gout incidence for serum urate bands between 4.00 mg/dL and 5.99 mg/dL (Table 3). There was a non-significant increased risk for serum urate bands between 6.00 mg/dL and 6.99 mg/dL, and significantly higher risk for serum urate > 7.00 mg/dL. In contrast, for females, compared to the referent baseline serum urate < 4.00 mg/dL, virtually all of the serum urate bands had an increased risk, although not all were statistically significant (Table 3). In all females, there was a significant increased risk of incident gout for baseline serum urate from 5.00–5.49 mg/dL. A similar pattern was observed for females who were > 50 years old at the start of the observation period, although for females ≤50 years of age, the risk of gout increased at a higher serum urate band (Supplementary Table 4).

### Best fit models at each time point

**Fig. 1:**
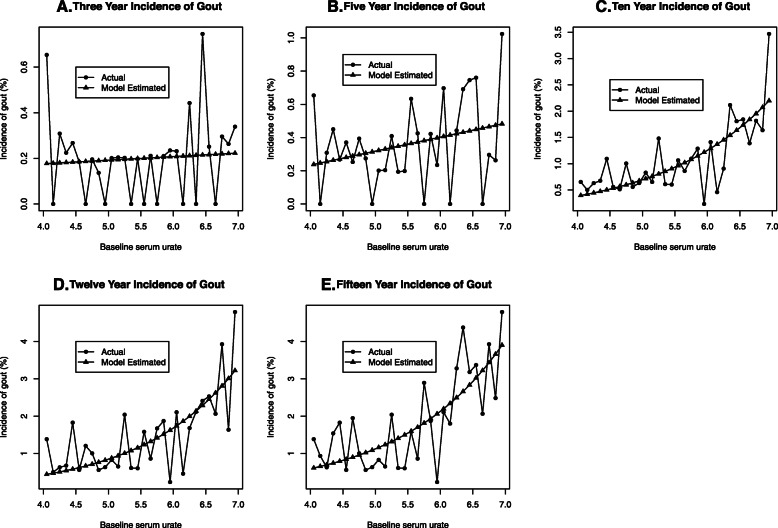
The incidence of gout observed in the cohort and the model predicted incidence (see methods) over each time period; **a**: 3 year, **b**: 5 year, **c**: 10 year, **d**: 12 year and **e**: 15 year

The relationship between baseline serum urate and incident gout in the early follow-up periods of three and 5 years was linear (see Eq. 1). However, an exponential component was required to best construct the 10, 12 and 15 year timepoints (see Eq. 2). The raw incidence figures and the incidence as predicted by the models is shown in Fig. 1. Model parameters for each timepoint in the combined gender group are shown in Supplementary Table 5.

1 $$ I=a+ bSU $$

Where I is incidence, a is the intercept, b is the slope and SU is serum urate. The model used for incident gout at the 10, 12 and 15 year time points are shown in Eq. 2.

2 $$ I= Exp\left(a+ bSU\right) $$

## Discussion

This longitudinal analysis of individual participant data shows that people with a baseline serum urate below the laboratory crystallization point of urate at temperature 37 °C can develop clinically evident gout over an extended observation time. While an elevated serum urate > 7.00 mg/dL is associated with substantially higher increased risk of incident gout, gout develops in 0.59–3.2% of those with lower serum urate levels (< 7.00 mg/dL). Given that the large majority of the population do not have serum urate levels > 7.00 mg/dL (even in contemporary obesogenic environments [18]), these findings have significance when considering population risk of gout.

The CPH analysis of the entire cohort (which adjusted for sex within the model) demonstrated that serum urate > 5.50 mg/dL is a risk factor for development of clinically evident gout over 15 years, compared with the referent baseline serum urate < 4.00 mg/dL. We did observe differences in the patterns of risk according to baseline serum urate band in males and females; in males there was higher risk for serum urate level bands between 6.00 mg/dL and 6.99 mg/dL, and significantly higher risk for serum urate > 7.00 mg/dL. In contrast, for females, virtually all of the serum urate level bands had an increased risk. The reason for this is unclear, but could reflect fundamental differences in the pathogenesis of gout between men and women, at least at lower urate levels. The difference seen in the female groups (less than and greater than 50 years of age) may well be related to menopausal status [20]. Estrogen is uricosuric and the loss of estrogen after menopause may explain the change in incident gout risk seen between the two female age groups [21].

The strengths of this data set include the large number of participants and the long follow-up time of 15 years. Limitations include analysis of a single serum urate level, which may not reflect testing in clinical practice. We did not include longitudinal medical information like kidney function, body mass index or medication use as we wanted to design models that could be used to provide an estimate of future incident gout without knowing what future changes in health might occur. The absence of covariables such as obesity and diuretic use mean that the model is not as accurate as it could have been. Many co-morbidities which impact on serum urate such as obesity, impaired kidney function and the metabolic syndrome are inter-related further complicating the construction of an accurate model. Although serum urate is generally a stable analyte, urate can change over time, particularly in the setting of changes in kidney function, medications, body weight, and post-menopause [9, 22, 23]. There was also variation in the ascertainment of gout across the cohorts, including some cohorts using self-report gout and so it is possible that incident gout may have been misclassified in some participants. However, the definitions of gout used in this study have equivalent accuracy compared to microscopically proven gout for use in epidemiological studies [24]. We were unable to adjust for competing risk of death in our analyses. The disconnect between the prevalence of hyperuricaemia (~ 20–25%) and incident gout (~ 5%) is an area which requires further investigation to identify other factors that promote clinically evident gout.

## Conclusions

In summary, although baseline serum urate of 5.50 mg/dL or more is a risk factor for development of gout, incident gout does occur over time in a small proportion of people with lower baseline serum urate levels. These data will enable the estimation of the effect of public health interventions to lower serum urate on the risk of subsequent incident gout.

## Supplementary Information

**Additional file 1: Supplementary Table 1.** Visit details, gout definitions, and participant characteristics for each cohort (Modified from ﻿Dalbeth N, et al. Ann Rheum Dis 2018;77:1048–1052.).

**Additional file 2: Supplementary Table 2.** Raw incidence of gout seen at timepoints 3, 5, 10, 12 and 15 years of follow-up in the exploratory analysis of the female cohort which was split into those who were less than or equal to 50 years of age at the time of the observation period and those who were greater than 50 years of age at the start of the observation period. Raw incidence and 95% confidence intervals are shown.

**Additional file 3: Supplementary Table 3.** Full adjusted (top panel) and unadjusted (lower panel) models.

**Additional file 4: Supplementary Table 4.** Cox proportional hazards models for the exploratory analysis of the female cohort which was split into those who were less than or equal to 50 years of age at the time of the observation period and those who were greater than 50 years of age at the start of the observation period The model was adjusted for the following covariables: age, ethnicity and original cohort.

**Additional file 5:Supplementary Table 5.** The details of the models estimating gout incidence for the entire cohort at timepoints 3, 5, 10, 12 and 15 years with equation, model parameters, standard error and 95% confidence intervals.

## Data Availability

The cohort data are available on application to the National Institutes of Health (NIH) Database of Genotypes and Phenotypes, 9000 Rockville Pike, Bethesda, Maryland 20892.

## Abbreviations

ARIC: Atherosclerosis Risk in Communities Study
CARDIA: Coronary Artery Risk Development in Young Adults Study
CPH: Cox proportional hazards
FHS: Framingham Heart Study
HR: Hazard ratio
IRB: Institutional Review Board
SD: Standard deviation
SU: Serum urate
