# Supplementary material for: Longitudinal development of incident gout from low-normal baseline serum urate concentrations: individual participant data analysis
Source: BMC Rheumatol. 2021 Aug 28;5:33. doi: 10.1186/s41927-021-00204-4 (PMC8399746; doi:10.1186/s41927-021-00204-4)
Supplement: Supplementary file 1 — Additional file 1: Supplementary Table 1. Visit details, gout definitions, and participant characteristics for each cohort (Modified from ﻿Dalbeth N, et al. Ann Rheum Dis 2018;77:1048–1052.). [file 41927_2021_204_MOESM1_ESM.docx]

**Supplementary Table 1. Visit details, gout definitions, and participant characteristics for each cohort (Modified from ﻿Dalbeth N, et al. Ann Rheum Dis 2018;77:1048–1052.)**

|  | | **ARIC** | **CARDIA** | **FHS Original** | **FHS Offspring** |
| --- | --- | --- | --- | --- | --- |
| **Description of cohort and setting** | Population-based cohort recruited in four sites in the US (Forsyth County, North Carolina; Jackson, Mississippi; suburban Minneapolis, Minnesota; and Washington County, Maryland) | | Population-based cohort recruited in four sites in the US (Birmingham, Alabama; Chicago, Illinois; Minneapolis, Minnesota; and Oakland, California) | Population-based cohort of residents in Framingham, Massachusetts, USA | Population-based second generation cohort of offspring in the Original FHS study |
| **Key inclusion criteria for cohort study** | Aged 45-64 at the time of enrolment into the cohort | | African American and White adults aged 18-30 at the time of enrolment into the cohort | Age 30-62 at the time of enrolment into the cohort, with no history of heart attack or stroke | Children of the Original FHS Cohort, or spouses if they had become pregnant with (or sired) two or more children by a participant in the Offspring Cohort |
| **Cohort examination number for serum urate (baseline)** | 1 | | 1 | 13 | 2 |
| **Year of baseline serum urate visit** | 1987 to 1989 | | 1985 to 1986 | 1972 to 1976 | 1979 to 1983 |
| **Cohort examination number for prevalent gout** | 4 | | 4, 5, 6 | 13 | 2 |
| **Cohort examination number/s for incident gout** | 4 | | 4, 5, 6 | 14-21 | 3, 4, 5, 6, 7 |
| **Duration of follow-up from baseline serum urate, years** | 9 | | 15 | 16 | 19 |
| **Definition of prevalent gout** | Gout diagnosis reported at examination 4 with onset prior to examination 1 date | | Gout diagnosis reported at examination 4, 5, 6, with onset prior to examination 1 date | “Clinical Diagnostic Impression: Gout: Yes” reported at examination 13 | “Clinical Diagnostic Impression: Gouty Arthritis: Yes” reported at examination 2 |
| **Definition of incident gout** | Gout diagnosis reported at examination 4 with onset after examination 1 date | | Gout diagnosis reported at examination 4, 5, or 6, with onset after examination 1 date | “Clinical Diagnostic Impression: Gout: Yes” reported at examinations 14-21, and not reported at examination 13 | “Clinical Diagnostic Impression: Gout: Yes” reported at examinations 3, 4, 5, 6, or 7, and “Clinical Diagnostic Impression: Gouty Arthritis: Yes” not reported at examination 2 |
| **Date of incident gout** | Calculated date of gout diagnosis | | Calculated date of gout diagnosis | Available for examination dates only | Available for examination dates only |
| **Total number of participants in dataset** | 10,775 | | 3,470 | 2,883 | 2,268 |
| **N with prevalent gout at baseline** | 403 (3.7%) | | 8 (0.2%) | 78 (2.7%) | 18 (0.8%) |
| **N included in incident gout analysis** | 10,372 | | 3,462 | 2,805 | 2,250 |
| **Male sex, n (%)*** | 4,558 (43.9%) | | 1,520 (43.9%) | 1,175 (41.9%) | 1,027 (45.6%) |
| **Mean (range) age baseline *** | All participants: 54 (45-64)  Men: 54 (45-64)  Women: 54 (45-64) | | All participants: 25 (17-34)  Men: 25 (17-34)  Women: 25 (17-34) | All participants: 66 (53-85)  Men: 65 (53-84)  Women: 66 (53-85) | All participants: 42 (17-67)  Men: 42 (18-67)  Women: 42 (17-67) |
| **Ethnicity, n (%)*** | White 8,203 (79.1%)  African American 2,169 (20.9%) | | White 1,871 (54.0%)  African American 1,591 (46.0%) | White 2,805 (100%) | White 2,216 (98.5%) |
| **Serum urate, mg/dL, mean (SD)*** | 5.8 (1.5) | | 5.2 (1.3) | 5.2 (1.3) | 4.8 (1.3) |
| **Number of incident gout events*** | 246 (2.4%) | | 48 (1.4%) | 129 (4.6%) | 72 (3.2%) |

*for those included in the incident gout analysis (those with prevalent gout excluded)
