# Supplementary material for: Longitudinal development of incident gout from low-normal baseline serum urate concentrations: individual participant data analysis
Source: BMC Rheumatol. 2021 Aug 28;5:33. doi: 10.1186/s41927-021-00204-4 (PMC8399746; doi:10.1186/s41927-021-00204-4)
Supplement: Supplementary file 2 — Additional file 2: Supplementary Table 2. Raw incidence of gout seen at timepoints 3, 5, 10, 12 and 15 years of follow-up in the exploratory analysis of the female cohort which was split into those who were less than or equal to 50 years of age at the time of the observation period and those who were greater than 50 years of age at the start of the observation period. Raw incidence and 95% confidence intervals are shown. [file 41927_2021_204_MOESM2_ESM.docx]

Supplementary Table 2: Raw incidence of gout seen at timepoints 3, 5, 10, 12 and 15 years of follow-up in the exploratory analysis of the female cohort which was split into those who were less than or equal to 50 years of age at the time of the observation period and those who were greater than 50 years of age at the start of the observation period. Raw incidence and 95% confidence intervals are shown.

|  | SU (mg/dL) | N | 3 Years | 5 Years | 10 Years | 12 Years | 15 Years |
| --- | --- | --- | --- | --- | --- | --- | --- |
|  |  |  | Incidence | Incidence | Incidence | Incidence | Incidence |
| Females ≤ 50 years old | < 4.00 | 1472 | 0.00% | 0.07% | 0.27% | 0.36% | 0.36% |
|  | 4.00 - 4.49 | 884 | 0.00% | 0.00% | 0.11% | 0.30% | 0.30% |
|  | 4.50 - 4.99 | 857 | 0.00% | 0.35% | 0.82% | 0.82% | 0.82% |
|  | 5.00 - 5.49 | 665 | 0.00% | 0.00% | 0.46% | 0.46% | 0.46% |
|  | 5.50 - 5.99 | 435 | 0.23% | 0.23% | 0.72% | 1.21% | 1.21% |
|  | 6.00 - 6.49 | 279 | 0.36% | 0.36% | 1.08% | 1.08% | 1.08% |
|  | 6.50 - 6.99 | 158 | 0.00% | 0.63% | 1.90% | 1.90% | 1.90% |
|  | 7+ | 212 | 0.00% | 0.94% | 7.22% | 8.84% | 8.84% |
|  |  |  |  |  |  |  |  |
| Females > 50 years old | < 4.00 | 816 | 0.13% | 0.13% | 0.41% | 0.41% | 0.71% |
|  | 4.00 - 4.49 | 721 | 0.43% | 0.57% | 1.34% | 1.34% | 2.50% |
|  | 4.50 - 4.99 | 932 | 0.00% | 0.11% | 0.23% | 0.68% | 0.68% |
|  | 5.00 - 5.49 | 902 | 0.45% | 0.68% | 1.39% | 1.39% | 1.39% |
|  | 5.50 - 5.99 | 702 | 0.14% | 0.29% | 1.04% | 1.04% | 1.04% |
|  | 6.00 - 6.49 | 548 | 0.00% | 0.00% | 1.20% | 2.48% | 2.48% |
|  | 6.50 - 6.99 | 392 | 0.00% | 0.26% | 2.01% | 2.01% | 2.01% |
|  | 7+ | 634 | 1.12% | 2.76% | 8.50% | 15.57% | 21.62% |
