# Supplementary material for: Longitudinal development of incident gout from low-normal baseline serum urate concentrations: individual participant data analysis
Source: BMC Rheumatol. 2021 Aug 28;5:33. doi: 10.1186/s41927-021-00204-4 (PMC8399746; doi:10.1186/s41927-021-00204-4)
Supplement: Supplementary file 3 — Additional file 3: Supplementary Table 3. Full adjusted (top panel) and unadjusted (lower panel) models. [file 41927_2021_204_MOESM3_ESM.docx]

Supplementary Table 3: Full adjusted (top panel) and unadjusted (lower panel) models

| Adjusted analysis | B | SE | Wald | df | p-value | HR | 95.0% CI for HR | |
| --- | --- | --- | --- | --- | --- | --- | --- | --- |
|  |  |  |  |  |  |  | Lower | Upper |
| COHORT (referent ARIC) |  |  | 58.740 | 3 | 0.000 |  |  |  |
| CARDIA | 0.456 | 0.395 | 1.330 | 1 | 0.249 | 1.578 | 0.727 | 3.423 |
| FHSOFFSP | 0.553 | 0.183 | 9.115 | 1 | 0.003 | 1.738 | 1.214 | 2.487 |
| FHSOrigi | 1.124 | 0.148 | 57.848 | 1 | 0.000 | 3.079 | 2.304 | 4.113 |
| SEX (Female) | -0.267 | 0.105 | 6.422 | 1 | 0.011 | 0.766 | 0.623 | 0.941 |
| AGE deciles (referent <30) |  |  | 13.703 | 6 | 0.033 |  |  |  |
| 30.0 - 39.0 | 0.579 | 0.332 | 3.050 | 1 | 0.081 | 1.784 | 0.932 | 3.417 |
| 40.0 - 49.0 | 1.297 | 0.399 | 10.577 | 1 | 0.001 | 3.656 | 1.674 | 7.987 |
| 50.0 - 59.0 | 1.375 | 0.404 | 11.610 | 1 | 0.001 | 3.955 | 1.793 | 8.722 |
| 60.0 - 69.0 | 1.376 | 0.415 | 11.009 | 1 | 0.001 | 3.958 | 1.756 | 8.922 |
| 70.0 - 79.0 | 1.476 | 0.461 | 10.262 | 1 | 0.001 | 4.374 | 1.773 | 10.788 |
| 80.0+ | 1.823 | 0.615 | 8.778 | 1 | 0.003 | 6.191 | 1.854 | 20.681 |
| Ethnicity (Caucasian) | -0.771 | 0.120 | 41.499 | 1 | 0.000 | 0.463 | 0.366 | 0.585 |
| Surate baseline (referent <4.0) |  |  | 419.473 | 7 | 0.000 |  |  |  |
| 4.00 - 4.49 | 0.671 | 0.343 | 3.840 | 1 | 0.050 | 1.957 | 1.000 | 3.830 |
| 4.50 - 4.99 | 0.357 | 0.347 | 1.057 | 1 | 0.304 | 1.429 | 0.724 | 2.821 |
| 5.00 - 5.49 | 0.502 | 0.335 | 2.241 | 1 | 0.134 | 1.652 | 0.856 | 3.186 |
| 5.50 - 5.99 | 0.957 | 0.317 | 9.094 | 1 | 0.003 | 2.603 | 1.398 | 4.847 |
| 6.00 - 6.49 | 1.308 | 0.308 | 18.013 | 1 | 0.000 | 3.700 | 2.022 | 6.770 |
| 6.50 - 6.99 | 1.657 | 0.305 | 29.506 | 1 | 0.000 | 5.242 | 2.883 | 9.531 |
| 7+ | 2.924 | 0.279 | 110.119 | 1 | 0.000 | 18.621 | 10.784 | 32.152 |
|  |  |  |  |  |  |  |  |  |
| Unadjusted analysis |  |  |  |  |  |  |  |  |
| Urate baseline (referent <4.0) |  |  | 527.023 | 7 | 0.000 |  |  |  |
| 4.00 - 4.49 | 0.703 | 0.342 | 4.235 | 1 | 0.040 | 2.021 | 1.034 | 3.948 |
| 4.50 - 4.99 | 0.449 | 0.346 | 1.687 | 1 | 0.194 | 1.567 | 0.796 | 3.084 |
| 5.00 - 5.49 | 0.614 | 0.332 | 3.415 | 1 | 0.065 | 1.848 | 0.963 | 3.544 |
| 5.50 - 5.99 | 1.075 | 0.312 | 11.877 | 1 | 0.001 | 2.930 | 1.590 | 5.400 |
| 6.00 - 6.49 | 1.435 | 0.301 | 22.798 | 1 | 0.000 | 4.200 | 2.330 | 7.569 |
| 6.50 - 6.99 | 1.782 | 0.296 | 36.271 | 1 | 0.000 | 5.942 | 3.327 | 10.613 |
| 7+ | 3.088 | 0.266 | 134.609 | 1 | 0.000 | 21.925 | 13.014 | 36.938 |
