# Supplementary material for: Longitudinal development of incident gout from low-normal baseline serum urate concentrations: individual participant data analysis
Source: BMC Rheumatol. 2021 Aug 28;5:33. doi: 10.1186/s41927-021-00204-4 (PMC8399746; doi:10.1186/s41927-021-00204-4)
Supplement: Supplementary file 4 — Additional file 4: Supplementary Table 4. Cox proportional hazards models for the exploratory analysis of the female cohort which was split into those who were less than or equal to 50 years of age at the time of the observation period and those who were greater than 50 years of age at the start of the observation period The model was adjusted for the following covariables: age, ethnicity and original cohort. [file 41927_2021_204_MOESM4_ESM.docx]

|  | Adjusted models | | | | | | |
| --- | --- | --- | --- | --- | --- | --- | --- |
|  | Serum Urate | Beta | SE | P | HR | 95% Lower CI | 95% Upper CI |
|  |  |  |  |  |  |  |  |
| Females ≤ 50 years old | Referant <4.0 mg/dL |  |  |  |  |  |  |
|  | 4.0-4.49 mg/dL | -0.784 | 0.807 | 0.331 | 0.46 | 0.09 | 2.22 |
|  | 4.5-4.99 mg/dL | 0.651 | 0.527 | 0.217 | 1.92 | 0.68 | 5.39 |
|  | 5.0-5.49 mg/dL | 0.174 | 0.639 | 0.785 | 1.19 | 0.34 | 4.17 |
|  | 5.5-5.99 mg/dL | 0.761 | 0.605 | 0.209 | 2.14 | 0.65 | 7.01 |
|  | 6.0-6.49 mg/dL | 0.641 | 0.715 | 0.370 | 1.90 | 0.47 | 7.72 |
|  | 6.5-6.99 mg/dL | 1.204 | 0.718 | 0.094 | 3.33 | 0.82 | 13.62 |
|  | ≥7 mg/dL | 2.419 | 0.509 | 0.000 | 11.23 | 4.15 | 30.43 |
|  |  |  |  |  |  |  |  |
| Females > 50 years old | Referant <4.0 mg/dL |  |  |  |  |  |  |
|  | 4.0-4.49 mg/dL | 1.436 | 0.580 | 0.013 | 4.20 | 1.35 | 13.09 |
|  | 4.5-4.99 mg/dL | 0.084 | 0.709 | 0.906 | 1.09 | 0.27 | 4.36 |
|  | 5.0-5.49 mg/dL | 1.307 | 0.581 | 0.024 | 3.70 | 1.18 | 11.54 |
|  | 5.5-5.99 mg/dL | 1.274 | 0.606 | 0.035 | 3.57 | 1.09 | 11.71 |
|  | 6.0-6.49 mg/dL | 1.445 | 0.619 | 0.020 | 4.24 | 1.26 | 14.28 |
|  | 6.5-6.99 mg/dL | 1.735 | 0.619 | 0.005 | 5.67 | 1.69 | 19.06 |
|  | ≥7 mg/dL | 3.391 | 0.529 | 0.000 | 29.69 | 10.52 | 83.79 |

Supplementary Table 4: Cox proportional hazards models for the exploratory analysis of the female cohort which was split into those who were less than or equal to 50 years of age at the time of the observation period and those who were greater than 50 years of age at the start of the observation period The model was adjusted for the following covariates: age, ethnicity and original cohort
