# Supplementary material for: Longitudinal development of incident gout from low-normal baseline serum urate concentrations: individual participant data analysis
Source: BMC Rheumatol. 2021 Aug 28;5:33. doi: 10.1186/s41927-021-00204-4 (PMC8399746; doi:10.1186/s41927-021-00204-4)
Supplement: Supplementary file 5 — Additional file 5:Supplementary Table 5. The details of the models estimating gout incidence for the entire cohort at timepoints 3, 5, 10, 12 and 15 years with equation, model parameters, standard error and 95% confidence intervals. [file 41927_2021_204_MOESM5_ESM.docx]

Supplementary Table 5: The details of the models estimating gout incidence for the entire cohort at timepoints 3, 5, 10, 12 and 15 years with equation, model parameters, standard error and 95% confidence intervals.

|  |  |  |  |  | **95% CI** | **95% CI** |
| --- | --- | --- | --- | --- | --- | --- |
| **Model** | **Timepoint** | **Parameter** | **Estimate** | **Standard Error** | **Lower bound** | **Upper bound** |
| a + bSU | 3 year | a | 0.0011 | 0.0022 | -0.0034 | 0.0056 |
|  |  | b | 0.0002 | 0.0004 | -0.0006 | 0.0010 |
| a + bSU | 5 year | a | -0.0010 | 0.0030 | -0.0071 | 0.0051 |
|  |  | b | 0.0008 | 0.0005 | -0.0003 | 0.0019 |
| Exp(a + bSU) | 10 year | a | -7.919 | 0.667 | -9.285 | -6.552 |
|  |  | b | 0.590 | 0.106 | 0.372 | 0.808 |
| Exp(a + bSU) | 12 year | a | -8.192 | 0.849 | -9.931 | -6.452 |
|  |  | b | 0.684 | 0.134 | 0.410 | 0.958 |
| Exp(a + bSU) | 15 year | a | -7.677 | 0.738 | -9.189 | -6.165 |
|  |  | b | 0.638 | 0.117 | 0.398 | 0.877 |
